# Supplementary material for: Kagome qubit ice
Source: Nat Commun. 2023 Feb 27;14:1105. doi: 10.1038/s41467-023-36760-1 (PMC9970994; doi:10.1038/s41467-023-36760-1)
Supplement: Supplementary file 1 — Supplementary Information [file 41467_2023_36760_MOESM1_ESM.pdf]

Kagome qubit ice: Supplementary Informations

Alejandro Lopez-Bezanilla[1], Jack Raymond[2], Kelly Boothby[2],
Juan Carrasquilla[3,4], Cristiano Nisoli[1,a,*], and Andrew D. King[2,b,*]

[1] *Theoretical Division, Los Alamos National Laboratory, Los Alamos, New Mexico 87545, USA*

[2] *D-Wave Systems, Burnaby, British Columbia, Canada, V5G 4M9, Canada*

[3] *Vector Institute, University of Toronto, Toronto, Ontario, M5G 1M1, Canada*

[4] *Department of Physics and Astronomy, University of Waterloo, Waterloo, Ontario, N2L 3G1, Canada*

[a] *cristiano@lanl.gov;* [b] *aking@dwavesys.com;* *and*

[*] *these two authors contributed equally.*

(Dated: January 17, 2023)

KAGOME ICE AND CLASSICAL TOPOLOGICAL ORDER: RELEVANT BACKGROUND

For the benefit of the reader unfamiliar with frustrated spin ice systems [1–6], we summarize in this section basic notions on kagome spin ice that are disseminated in various publications [7–12], adding also material that facilitates the reading of the main text.

Kagome spin ice is a set of binary variables, or Ising spins, arranged along the edges of a hexagonal lattice. The graph of the center of the edges of a hexagon—the "dual" graph—is a kagome lattice, hence the name. Among other names there is honeycomb spin ice and hexagonal spin ice.

In the simplest model all the spins impinging in a vertex interact with the same coupling, thus leading to the Hamiltonian of Eq. (1) in main text. This leads to a ground state in which all the vertices are minimally frustrated, with two moments pointing in and one pointing out, or vice-versa. The ground state thus excludes vertices where all three moments point in or all three point out at the same time. In other words, defining the charge of a vertex as number of spins pointing in minus those pointing out, the ground state of the kagome ice at the nearest neighbor coupling consist of vertices of charge ±1 only, excluding those of charge ±3. This manifold has an extensive degeneracy of entropy per spin $s_{\text{Ice-I}} \simeq (1/3)\ln(9/2) = 0.501$ [7] (this number is not computed exactly but obtained à la Pauling [13] and it is very close to the numerical results from Monte Carlo simulations). Thus, the entropy of the Ice-I manifold is quite close to the purely random entropy per spin $s_\infty = \ln 2 \simeq 0.693$.

However, in nano-scopic realizations [2, 4, 14–18], magnetic moments interact as dipoles, and at a long range. It has been shown theoretically [8] and via Monte Carlo simulations [8, 9] that a multipole expansion leads to an interaction among vertices that is of the Coulomb kind in the vertex charge, as the first correction to the nearest neighbor coupling. This in turn leads to a transition (in the Ising class) to a charge-ordered, yet spin-disordered manifold. In the charge-ordered state, because the hexagonal lattice is bipartite, alternating vertices have opposite charge, forming an ionic crystal.

This manifold, called Ice-II, is still spin-disordered. In fact its disorder can be mapped to the dimer cover model on a hexagonal lattice in the following way. Consider the ordered state in which all A vertices have charge -1 and all the B vertices have charge $+1$. Then, each A vertex has one and only one spin pointing in, and each B vertex has one and

only one spin pointing out, which we call a minority spin. It follows that each A vertex is connected by that minority spin to a B vertex and the two are coupled by sharing a minority spin. Because there is only one minority spin for each vertex, and because each vertex has one minority spin, minority spins are dimers of the hexagonal lattice. Then, the degeneracy of a dimer model on a bipartite lattice can be computed exactly with the method of Kasteleyn [19, 20]. Alternatively, one can then map the dimer model to a frustrated Ising model on the triangular lattice, as explained in the Supplementary Information of Ref. [21]. Either way, one obtains an entropy per spin $s_{\text{Ice-II}} = 0.155 \ln 2 \simeq 0.107$, considerably reduced with respect to the Ice-I phase, but still not zero.

That Ice-II is a topological phase is shown in Ref. [10]. The fact that it is equivalent to a dimer covering model should, whose topological properties are well known, should already suffice. In particular, one can partition its space into topological sectors [22–24].

While notions of topological orders originated in quantum mechanics, the late C. Henley argued convincingly [25, 26] in favor of a classical notion of topological order in systems that, as dimer coverings, admit a height formalism: "topological order (..) is meaningful in a purely classical model (...) Indeed, I would suggest that the subject of topological order skipped over more elementary examples, owing to historical accident" [25]. And after all, partitions into topological sectors of quantum dimer models are based on classical dimer configurations. In this view, topological order in a classical system points to the lack of an order parameter à la Landau, the partition of the phase space into topological sector, and the fact that only cooperative updates along a loop have no energy cost. The latter statement implies that in realistic systems, the kinetics proceeds through the creation, annihilation and propagation of fractionalized excitations [25], of which monopoles in spin ice as a paradigmatic example [27].

This is also what happens in the Ice-II phase. Because each single spin flip must alter the charge distribution, only flips of entire loops of head-to-toe spins are un-gapped. Alternatively, fractionalized excitations such as the ones described in the main text drive the kinetics, and conserve a topological charge. In pyrochlore and square spin ice the topological charge is proportional to the magnetic charge, whereas in the Ice-II phase of kagome ice it is not.

The topological structure of the Ice-II phase has motivated experimental attempts to reach it. These were successful in showing at least the formation of crystallites of charges [28]

whose size could later be controlled via skillful honing of the magnetic alloy used in fabrication [29] while signatures of critical slowing down corresponding to a possible transition to the Ice-II phase were detected via muon spectroscopy [30]. In nanoscopic magnets the phase proves hard to reach because it is driven by the weak charge-charge interaction of the vertices.

In this work we induce the Ice-II phase via field, rather than by long-range interaction, as the latter is inaccessible to a quantum annealer, whereas the former, accessible to a quantum annealer, is inaccessible to a nanomagnetic realization. Because of the Zeeman energy term $-hs_i$, the energies for B vertices of charge $q = \pm 1, \pm 3$ are

$$\begin{aligned} \epsilon_{+1} &= -J - h/2 \\ \epsilon_{-1} &= -J + h/2 \\ \epsilon_{+3} &= 3J - 3h/2 \\ \epsilon_{-3} &= 3J + 3h/2, \end{aligned} \tag{1}$$

which are plotted in Fig. 2 of the Main Text.

There are differences between a thermal phase and a field-induced phase. In a thermal phase transition there is a spontaneous symmetry breaking absent in the field-induced one. However, the study of the transition is not the aim of this work. Rather, we seek to show the topologically protected nature of the kinetics. This does not depend on how the phase is obtained, but merely follows from the constraints of the phase. Nonetheless, the reader should keep in mind that the fractionalized excitations would be different in the thermally induced Ice-II phase: violations of the ice rule, admissible in our case, would not be present, and the only excitations would consist of violations of charge order.

Additional data

In **Supplementary Fig. 1** we show additional examples of kinetics. Note that at $h/J = 0$ the pseudo-ice rule has a degenerate ground state for each vertex at charge $+1$ and -1, so neither is displayed as an excitation. For very small $h/J = 0.05$, this degeneracy is lifted slightly, and ice rule violations are abundant.

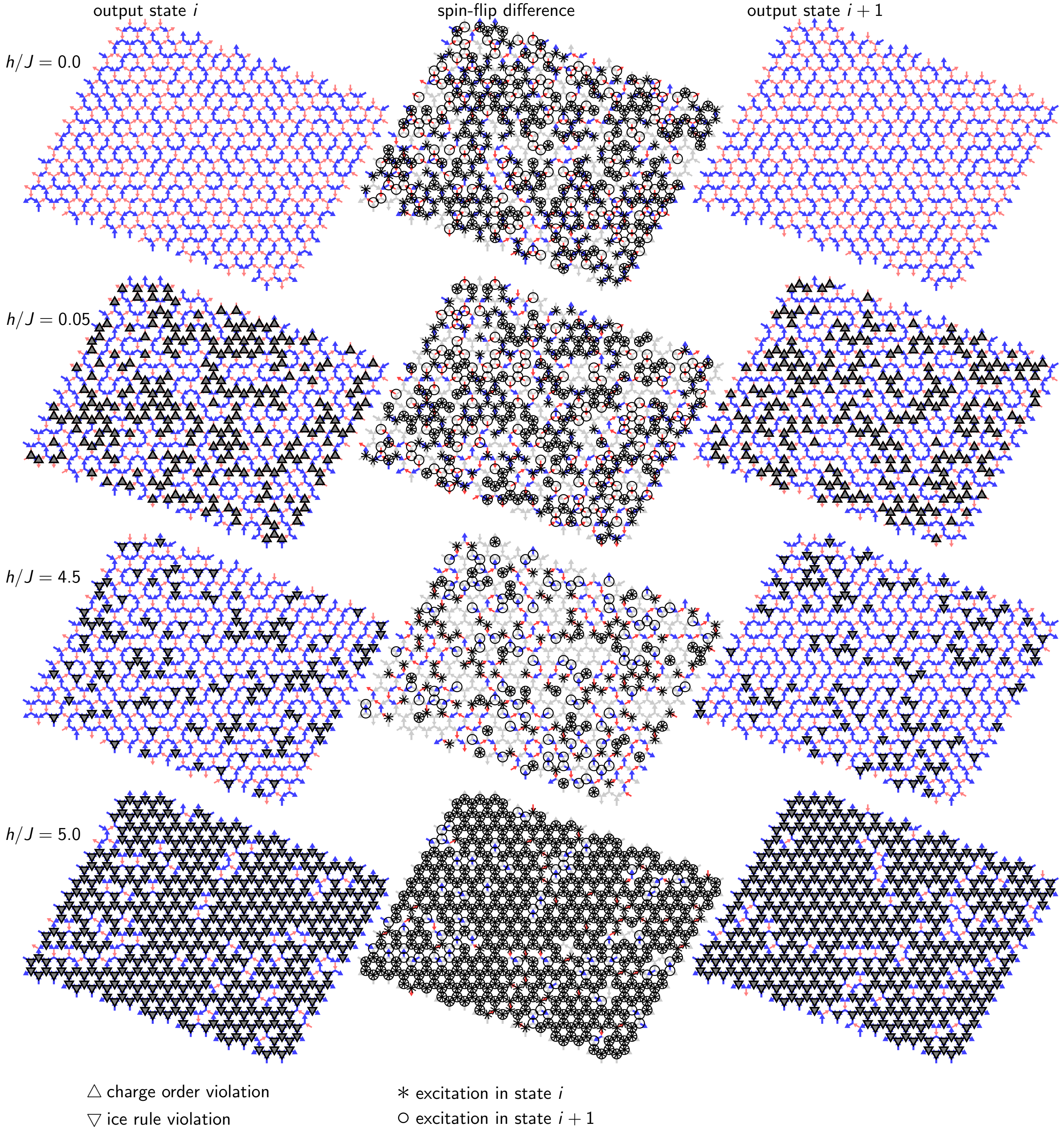

Supplementary Fig. 1: **Additional examples of kinetics.** Analogous to Fig. 4, shown are two consecutive states for values of h/J at and near the Ice-I phase ($h/J = 0$) and near the polarizing transition ($h/J \approx 4.5$).

[1] Balents, L. Spin liquids in frustrated magnets. *Nature* **464**, 199–208 (2010).

[2] Nisoli, C., Moessner, R. & Schiffer, P. Colloquium : Artificial spin ice: Designing and imaging magnetic frustration. *Reviews of Modern Physics* **85**, 1473–1490 (2013).

[3] Bramwell, S. T. & Harris, M. J. The history of spin ice. *Journal of Physics Condensed Matter* **32** (2020).

[4] Skjærvø, S. H., Marrows, C. H., Stamps, R. L. & Heyderman, L. J. Advances in artificial spin ice. *Nature Reviews Physics* **2**, 13–28 (2020).

[5] Henley, C. L. The "Coulomb Phase" in Frustrated Systems. *Annual Review of Condensed Matter Physics* **1**, 179–210 (2010).

[6] Henley, C. L. Relaxation time for a dimer covering with height representation. *Journal of statistical physics* **89**, 483–507 (1997).

[7] Wills, A., Ballou, R. & Lacroix, C. Model of localized highly frustrated ferromagnetism: The kagomé spin ice. *Physical Review B* **66**, 144407 (2002).

[8] Möller, G. & Moessner, R. Magnetic multipole analysis of kagome and artificial spin-ice dipolar arrays. *Phys. Rev. B* **80**, 140409 (2009).

[9] Chern, G.-W., Mellado, P. & Tchernyshyov, O. Two-stage ordering of spins in dipolar spin ice on the kagome lattice. *Phys. Rev. Lett.* **106**, 207202 (2011).

[10] Macdonald, A. J., Holdsworth, P. C. W. & Melko, R. G. Classical topological order in kagome ice. *Journal of Physics: Condensed Matter* **23**, 164208 (2011).

[11] Raban, V., Suen, C., Berthier, L. & Holdsworth, P. Multiple symmetry sustaining phase transitions in spin ice. *Physical Review B* **99**, 224425 (2019).

[12] Lhotel, E., Jaubert, L. D. & Holdsworth, P. C. Fragmentation in frustrated magnets: A review. *Journal of Low Temperature Physics* **201**, 710–737 (2020).

[13] Pauling, L. The Structure and Entropy of Ice and of Other Crystals with Some Randomness of Atomic Arrangement. *Journal of the American Chemical Society* **57**, 2680–2684 (1935).

[14] Qi, Y., Brintlinger, T. & Cumings, J. Direct observation of the ice rule in an artificial kagome spin ice. *Physical Review B* **77**, 1–4 (2008).

[15] Rougemaille, N. *et al.* Artificial kagome arrays of nanomagnets: A frozen dipolar spin ice. *Phys. Rev. Lett.* **106**, 057209 (2011).

[16] Gartside, J. C. *et al.* Realization of ground state in artificial kagome spin ice via topological defect-driven magnetic writing. *Nature Nanotechnology* **13**, 53–58 (2018).

[17] Chioar, I. A., Rougemaille, N. & Canals, B. Ground-state candidate for the classical dipolar kagome ising antiferromagnet. *Phys. Rev. B* **93**, 214410 (2016).

[18] Canals, B. *et al.* Fragmentation of magnetism in artificial kagome dipolar spin ice. *Nature Communications* **7**, 11446 (2016).

[19] Kasteleyn, P. W. The statistics of dimers on a lattice: I. the number of dimer arrangements on a quadratic lattice. *Physica* **27**, 1209–1225 (1961).

[20] Kasteleyn, P. W. Dimer statistics and phase transitions. *Journal of Mathematical Physics* **4**, 287–293 (1963).

[21] Lammert, P. E. *et al.* Direct entropy determination and application to artificial spin ice. *Nat. Phys.* **6**, 786–789 (2010).

[22] Rokhsar, D. S. & Kivelson, S. A. Superconductivity and the quantum hard-core dimer gas. *Physical review letters* **61**, 2376 (1988).

[23] Moessner, R., Sondhi, S. L. & Chandra, P. Phase diagram of the hexagonal lattice quantum dimer model. *Physical Review B* **64**, 144416 (2001).

[24] Misguich, G., Serban, D. & Pasquier, V. Quantum dimer model on the kagome lattice: Solvable dimer-liquid and ising gauge theory. *Physical review letters* **89**, 137202 (2002).

[25] Henley, C. L. Classical height models with topological order. *Journal of Physics: Condensed Matter* **23**, 164212 (2011).

[26] Lamberty, R. Z., Papanikolaou, S. & Henley, C. L. Classical topological order in abelian and non-abelian generalized height models. *Physical review letters* **111**, 245701 (2013).

[27] Castelnovo, C. & Chamon, C. Topological order and topological entropy in classical systems. *Physical Review B* **76**, 174416 (2007).

[28] Zhang, S. *et al.* Crystallites of magnetic charges in artificial spin ice. *Nature* **500**, 553–557 (2013).

[29] Drisko, J., Daunheimer, S. & Cumings, J. Fepd 3 as a material for studying thermally active artificial spin ice systems. *Physical Review B* **91**, 224406 (2015).

[30] Anghinolfi, L. *et al.* Thermodynamic phase transitions in a frustrated magnetic metamaterial. *Nature communications* **6** (2015).
